# Effectiveness of acupuncture combined with donepezil for Alzheimer’s disease: A systematic review and meta-analysis

**DOI:** 10.1097/MD.0000000000042651

**Published:** 2025-06-06

**Authors:** Yue Wu, Yijun Zhan, Wenyan Zhu, Jian Pei

**Affiliations:** a Department of Traditional Chinese Medicine, Ruijin Hospital, Shanghai Jiao Tong University School of Medicine, Shanghai, China; b Department of Acupuncture, Longhua Hospital, Shanghai University of Traditional Chinese Medicine, Shanghai, China.

**Keywords:** acupuncture, Alzheimer's disease, donepezil, meta-analysis

## Abstract

**Background:**

The incidence of Alzheimer's disease (AD) is gradually increasing in an aging society, imposing a heavy burden on society. Current studies have found that acupuncture therapy combined with donepezil has a good clinical effect in treating AD. We plan to conduct a systematic review and meta-analysis to evaluate the effectiveness of acupuncture combined with donepezil in AD patients.

**Methods:**

Eight databases were searched for randomized controlled trials (RCTs) using acupuncture in combination with donepezil for the treatment of AD, from the establishment of the database to October 1st, 2023. The clinical efficacy rate, Mini-Mental State Examination, AD Assessment Scale for Cognitive Capacity, Skill Level on Activities of Daily Living, Montreal Cognitive Assessment, Behavioral Pathology in AD Rating Scale, and adverse events were mainly used to evaluate the outcomes. RevMan 5.4.1 software was used to evaluate the quality of the included studies and perform a meta-analysis.

**Results:**

A total of 12 RCTs were included. Meta-analysis showed that acupuncture combined with donepezil seemed to be more effective than donepezil monotherapy for treatment of AD in improving the clinical efficacy rate (relative risk = 1.35; 95% confidence interval [CI]: 1.17–1.56; Z = 4.10; *P* < .0001), the Mini-Mental State Examination score (mean difference [MD] = 3.28; 95% CI: 1.81–4.75; Z = 4.37; *P* < .0001), and the Montreal Cognitive Assessment score (MD = 6.04; 95% CI: 4.76–7.32; Z = 9.23; *P* = .00001), while reducing the AD Assessment Scale for Cognitive Capacity score (MD = -3.57; 95% CI: -3.94 to -3.20; Z = 18.91; *P* < .00001), the Skill Level on Activities of Daily Living score (MD = -2.52; 95% CI: -4.05 to -0.99; Z = 3.23; *P* = .001), and the Behavioral Pathology in AD Rating Scale score (MD = -4.04; 95% CI: -4.58 to -3.50; Z = 14.64; *P* < .00001).

**Conclusions:**

Acupuncture combined with donepezil is an effective treatment which can improve cognitive ability and quality of life for AD patients. However, it is imperative to conduct more large-scale and high-quality RCTs in order to establish more definitive conclusions regarding this therapeutic approach in the future.

## 1. Introduction

Alzheimer's disease (AD), the most prevalent type of dementia characterized by memory loss, cognitive impairment, and personality changes, is a common neurodegenerative disease.^[1]^ Age is one of the greatest risk factors for AD, with the incidence and prevalence increasing exponentially after age 65, doubling roughly every 5 years.^[2,3]^ It is projected that over one-sixth of the world’s population will be aged 60 or above by 2030 with the rapid progression of global population aging; furthermore, this proportion is expected to double again by 2050.^[4]^ A gradual increase in both incidence and mortality rates is associated with age-related AD concurrently, and AD has emerged as the 7th leading cause of death worldwide.^[5]^ The prevention and treatment strategies for AD have thus garnered significant attention within society due to its escalating prevalence and impact on public health.

There is currently a lack of effective remedies for AD. The recommended primary medications endorsed by authorities primarily encompass acetylcholinesterase inhibitors and excitatory amino acid receptor antagonists, which can enhance cognitive function in patients but are unable to reverse the progression of the disease.^[6]^ Donepezil stands out as an exemplary acetylcholinesterase inhibitor among these due to its commendable efficacy in improving cognitive function and quality of life among individuals with mild to moderate AD.^[7]^ However, the incidence of adverse events in donepezil increased with increasing dose,^[8]^ numerous severe AD patients receiving high-dose donepezil treatment discontinued therapy due to a range of adverse events.^[9]^ Nevertheless, it should be noted that its application as a standalone treatment modality may have certain limitations based on clinical observations.^[10]^

Acupuncture is one of the representative non-pharmacological interventions, which has been proved to be effective in improving the cognitive function of AD patients in previous studies.^[11]^ It was showed in basic research that acupuncture may intervene the occurrence and development of AD through multiple mechanisms to achieve therapeutic effect.^[12]^ In certain system reviews, it has been confirmed that the acupuncture and acupuncture-related therapies are effective to improve the cognitive function of patients with mild to moderate AD,^[13]^ and the combination of acupuncture and drug therapy may confer greater benefits compared to drug therapy alone.^[14,15]^

Our previous study had demonstrated that the combination of acupuncture with donepezil in the treatment of AD superior improvements in cognitive function compared to donepezil monotherapy.^[16]^ However, the clinical efficacy of acupuncture combined with donepezil in AD patients remains undetermined. Therefore, it is imperative to elucidate the effectiveness of acupuncture combined with donepezil compared to donepezil monotherapy in the clinical application of AD, aiming to optimize the advantages of traditional Chinese medicine treatment and thereby enhance the overall therapeutic outcome for AD.

This study aims to compile randomized controlled trials (RCTs) results comparing acupuncture combined with donepezil versus donepezil monotherapy for AD treatment and conduct a meta-analysis. The objective is to identify an approach for integrating traditional Chinese medicine with Western medicine, combining medication with non-pharmacological interventions for AD, and provide evidence-based clinical treatment strategies and a solid foundation for future clinical applications.

## 2. Materials and methods

### 2.1. Registration

This meta-analysis was conducted in accordance with the Preferred Reporting Items for Systematic Reviews and Meta-Analyses (PRISMA) statement.^[17]^ The study has been registered on PROSPERO (CRD42023473205; https://www.crd.york.ac.uk/prospero/).

### 2.2. Selection criteria

RCTs that satisfied the diagnostic criteria for AD were incorporated, including (1) the Diagnostic and Statistical Manual of Mental Disorder IV (DSM-IV)^[18]^; (2) the National Institute on Aging-Alzheimer Association^[19]^; (3) the National Institute of Neurological and Communicative Disorder and Stroke: AD and Related Disorder Association^[20]^; (4) the International Classification of Disease version 10 (ICD-10)^[21]^; (5) the Chinese Guidelines for the Diagnosis and Treatment of Dementia and Cognitive Impairment^[22]^; (6) the Chinese Criteria for Classification and Diagnosis of Mental Disorders (Senile dementia).^[23]^

Participants in the experimental group received a comprehensive treatment, which included acupuncture therapy (scalp acupuncture, body acupuncture, electroacupuncture, fire needling, acupoint thread embedding, and acupoint injection, etc), in conjunction with donepezil. The control group solely received donepezil. The study yielded at least 1 distinct outcome, including the clinical efficacy rate, Mini-Mental State Examination (MMSE), AD Assessment Scale for Cognitive Capacity (ADAS-cog), Skill Level on Activities of Daily Living (ADL), Montreal Cognitive Assessment (MoCA), Behavioral Pathology in AD Rating Scale (BEHAVE-AD), and adverse events.

Some studies were excluded including animal trials, case reports, reviews, experiential presentations, conference proceedings, academic dissertations, protocols for RCTs, non-RCTs, repeatedly published literature, studies with unavailable full-text versions or vague data, and those lacking expected outcomes. Participants who had received treatments other than acupuncture combined with donepezil were also excluded.

### 2.3. Search strategy

An extensive search was conducted across 8 databases without language restrictions, to identify eligible studies from base-building to October 1st, 2023. This included 4 international databases (PubMed, Cochrane Library, Embase, and Web of Science) and 4 Chinese databases (China National Knowledge Infrastructure (CNKI), China Science and Technology Journal Database (VIP), Chinese BioMedical Literature Database (CBM), and Wanfang Data).

Two reviewers independently employed the PICOS (population, interventions, comparators, outcomes, study design) framework to filter and validate articles, leveraging MESH terms and free words for database queries, and adopting distinct database modification strategies. A third reviewer will be commissioned to appraise the entire manuscript when disputes emerge, and disagreements will be settled through a collective discussion.

Take the search strategy in PubMed as an example:

(1) AD [Mesh] OR Dementia [Mesh]
(2) AD [Title/Abstract] OR AD [Title/Abstract] OR AD [Title/Abstract] OR Dementia [Title/Abstract]
(3) 1 OR 2
(4) Acupuncture [Mesh] OR Acupuncture therapy [Mesh]
(5) Acupuncture* [Title/Abstract] OR acupuncture therapy [Title/Abstract] OR needle therapy [Title/Abstract] OR electroacupuncture* [Title/Abstract] OR electro-acupuncture [Title/Abstract] OR acupuncture point* [Title/Abstract] OR acupoint* [Title/Abstract] OR acupoint therapy [Title/Abstract] OR needl* [Title/Abstract] OR needle warming therapy [Title/Abstract] OR ear acupuncture [Title/Abstract] OR auricular acupuncture [Title/Abstract] OR auriculotherapy [Title/Abstract] OR hydro-acupuncture [Title/Abstract] OR acupoint injection [Title/Abstract] OR thread embedding [Title/Abstract] OR fire needling [Title/Abstract] OR scalp acupuncture [Title/Abstract] OR body acupuncture [Title/Abstract] OR abdominal acupuncture [Title/Abstract]
(6) 4 OR 5
(7) Clinical trials as topic [Mesh] OR clinical trial [Publication Type] [Mesh]
(8) Randomized controlled trial [Title/Abstract] OR RCT [Title/Abstract] OR random* [Title/Abstract]
(9) 7 OR 8
(10) 3 AND 6 AND 9

### 2.4. Data extraction

Two reviewers independently extracted the data and collected it into a spreadsheet. If discrepancies arose, a third reviewer would resolve them. The following information were extracted from each study: (1) first author and publication year; (2) diagnostic criteria; (3) sample size, average age, and average course of disease in both experimental and control groups; (4) intervention measures implemented, including treatment frequency, duration, and total treatment course; (5) anticipated outcomes and (6) adverse events.

### 2.5. Assessment of risk of bias in included studies

The risk of bias in studies was evaluated by 2 reviewers utilizing the specific questions based on the Cochrane risk-of-bias tool, followed by assigning ratings to these domains (random sequence generation, allocation concealment, blinding of participants and personnel, blinding of outcome assessment, incomplete outcome data, selective reporting, and other biases) as low, high, or unclear risk of bias. All disputes arising during the risk of bias assessment were resolved through internal discussions among the reviewers.

### 2.6. Data synthesis and statistical analysis

RevMan 5.4.1 software was utilized to combine the mean differences (MDs) for continuous outcomes and relative risks (RRs) for dichotomous outcomes, along with their corresponding 95% confidence intervals (CIs). Heterogeneity among studies was assessed using the Q test (Chi-square test) and the I^2^ statistic. If I^2^ ≤ 50%, it indicated a low level of heterogeneity, thus requiring the application of a fixed-effects model for analysis. Conversely, if I^2^ > 50%, it suggested the presence of heterogeneity among the studies, necessitating the use of a random-effects model for analysis. Subgroup analysis was conducted for different kinds of acupuncture therapies implemented in the experimental groups to investigate heterogeneity. Sensitivity analysis was performed using the leave-one-out method and conducted to ensure result stability when deemed necessary. In case of a sufficient number of studies, potential publication bias would be explored through funnel plotting. A difference was considered statistically significant if *P* < .05.

### 2.7. Ethics

No further ethical clearance is necessary as it is a secondary study based on published literature.

## 3. Results

### 3.1. Study search findings and study characteristics

**Figure 1.**
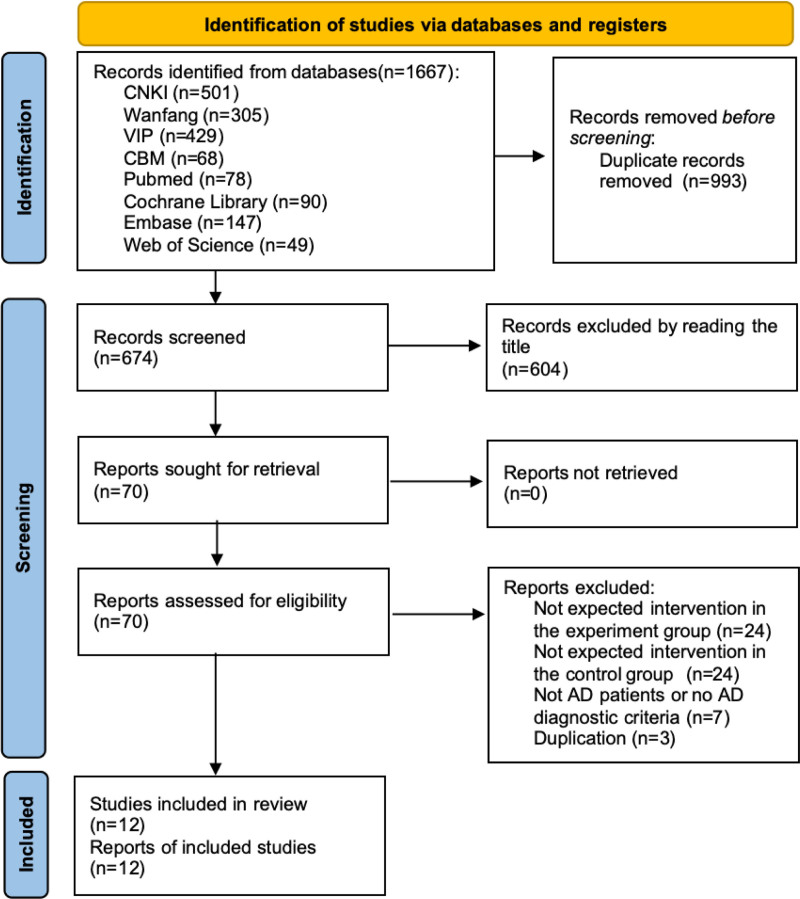
PRISMA flow diagram of screening process. PRISMA = preferred reporting items for systematic reviews and meta-analyses.

There were 12 studies^[24–35]^ met the inclusion criteria. The selection process is shown in Figure 1. The studies encompassed a total of 893 participants, with 444 allocated to the experimental group and 449 assigned to the control group. All studies were conducted in China and published within the timeframe of 2013 to 2023. Table 1 presents the characteristics of the included studies and outlines the features of the interventions utilized.

**Table 1:** Summary of included studies.

Included trial	Sample size (E/C)	Diagnostic criteria	Course of disease (year)		Age (year)		Intervention		Frequency and dose		Course of treatment	Intervention retention time (min)	Outcomes	Safety
			E	C	E	C	E	C	E	C				
Wang et al (2023)^[24]^	28/29	ICD-10	–	–	78.47 ± 5.10	77.40 ± 4.83	Body acupuncture + Donepezil	Donepezil	tiw + 5 mg qd	5 mg qd	12 w	40 minutes	MMSE;	No adverse events.
Liu et al (2022)^[25]^	64/64	NIA-AA	2.96 ± 0.85	2.97 ± 0.84	62.40 ± 3.39	62.52 ± 3.56	Electroacupuncture + Donepezil	Donepezil	qd + 5–10 mg qd	5–10 mg qd	8 w	40 minutes	Clinical efficacy rate; ADAS-cog; BEHAVE-AD;	–
Han et al (2022)^[26]^	32/34	Chinese Criteria for Classification and Diagnosis of Mental Disorders (Senile dementia)	3.82 ± 1.08	3.89 ± 1.21	66.61 ± 3.14	66.59 ± 3.21	Fire needling + scalp and body acupuncture + Donepezil	Donepezil	tiw + 5 times per week + 5 mg qd	5 mg qd	24 w	Immediate; 30 minutes	Clinical efficacy rate;	–
Zhou et al (2021)^[27]^	31/33	NINCDS-ADRDA	3.2	3.1	75.1 ± 7.0	75.8 ± 6.4	Electroacupuncture + Donepezil	Donepezil	tiw + 5 mg qd	5 mg qd	8 w	20 minutes	Clinical efficacy rate; ADAS-cog; MMSE;	3 mild adverse reactions in the experimental group; 2 mild adverse reactions in the control group (none required any treatment intervention).
Yang et al (2021)^[28]^	27/27	Chinese Guidelines for the Diagnosis and Treatment of Dementia and Cognitive Impairment	2.4 ± 0.7	2.3 ± 0.7	65 ± 12	65 ± 12	Abdominal acupoint thread embedding + Donepezil	Donepezil	once per 10 d + 5 mg qd	5 mg qd	8 w	Retentive	ADAS-cog; MMSE; ADL;	No adverse events.
Wang et al (2020)	31/30	NINCDS-ADRDA	3	2.5	72.74 ± 8.36	75.77 ± 7.03	Electroacupuncture + Donepezil	Donepezil	tiw + dose was the same as before enrollment	dose was the same as before enrollment	8 w	20 minutes	Clinical efficacy rate; ADAS-cog; MMSE;	–
Xia et al (2020)^[30]^	30/30	NIA-AA	4.32 ± 2.23	4.58 ± 2.12	61 ± 8	62 ± 7	Electroacupuncture + Donepezil	Donepezil	qd + 5–10 mg qd	5–10 mg qd	8 w	40 minutes	ADAS-cog; MoCA;	No adverse events.
Chen et al (2020)^[31]^	50/50	NINCDS-ADRDA	4.02 ± 0.11	3.84 ± 0.19	73.16 ± 7.69	72.06 ± 6.97	Acupoint injection + Donepezil	Donepezil	qod + 5–10 mg qd	5–10 mg qd	24 w	1–3 minutes	Clinical efficacy rate;	–
Zhang et al (2019)	46/46	ICD-10	–	–	72.6 ± 9.2	71.7 ± 8.7	Fire needling + Donepezil	Donepezil	qw + 5–10 mg qd	5–10 mg qd	12 w	Immediate	Clinical efficacy rate; MMSE; ADL;	2 cases of nausea or vomiting in the experimental group (4.35%); 8 cases of adverse events including 2 cases of diarrhea, 5 cases of nausea or vomiting, and 1 case of insomnia in the control group(17.39%). A significant difference existed between the 2 groups regarding their incidence rates of adverse events (*P* < .05).
Chen et al (2018)^[33]^	48/48	NIA-AA	3.42 ± 0.73	3.29 ± 0.68	74.36 ± 5.47	75.13 ± 5.81	Scalp and body acupuncture + Donepezil	Donepezil	bid + 5 mg qd	5 mg qd	12 w	1 minutes	Clinical efficacy rate; MMSE; ADL; BEHAVE-AD;	–
Wang et al (2014)^[34]^	27/28	DSM-Ⅳ	0.42 ± 0.09	0.48 ± 0.05	70.3 ± 8.0	70.7 ± 9.1	Scalp acupuncture + Donepezil	Donepezil	qd + 5–10 mg qd	5–10 mg qd	20 d	30 minutes	Clinical efficacy rate; ADAS-cog; MMSE;	–
Yin et al (2013)^[35]^	30/30	NINCDS-ADRDA	–	–	–	–	Scalp acupuncture + Donepezil	Donepezil	qd + 5 mg qd	5 mg qd	12 w	45 minutes	MMSE; ADL;	Conducted a comprehensive safety evaluation encompassing neurological examination as well as vital sign and laboratory assessments but did not provide final results for these evaluations.

Data presented as mean ± standard deviation.

bid = twice a day, C = control groups, d = day, DSM-IV = the Diagnostic and Statistical Manual of Mental Disorder IV, E = experimental groups, min = minute, ICD-10 = the International Classification of Disease version 10, NIA-AA = the National Institute on Aging-Alzheimer Association, NINCDS-ADRDA = the National Institute of Neurological and Communicative Disorder and Stroke: Alzheimer’s Disease and Related Disorder Association, qd = once a day, qw = once a week, tiw = 3 times a week, w = week.

#### 3.1.1. Diagnostic crteria

Of the 12 studies, 4^[27,29,31,35]^ adopted the diagnostic criteria of the National Institute of Neurological and Communicative Disorder and Stroke: AD and Related Disorder Association in 2011, and 3^[25,30,33]^ adopted the diagnostic criteria published by National Institute on Aging-Alzheimer Association. Two studies^[24,32]^ were diagnosed using ICD-10 criteria. One^[34]^ study was diagnosed using DSM-IV criteria. One^[28]^ used Chinese Guidelines for the Diagnosis and Treatment of Dementia and Cognitive Impairment. One study^[26]^ used Chinese Criteria for Classification and Diagnosis of Mental Disorders (Senile dementia).

#### 3.1.2. Interventions

In all the experimental groups included in the studies, 4^[24,33–35]^ reported manual acupuncture (including scalp acupuncture and body acupuncture), 4^[25,27,29,30]^ reported electroacupuncture, 2^[26,32]^ reported fire needling, while abdominal acupoint thread embedding^[28]^ and acupoint injection^[31]^ were each reported once. The course of treatment ranged from 20 days to 24 weeks. All control groups exclusively received donepezil as their treatment regimen, whereas the experimental groups combined acupuncture therapy with equivalent doses of donepezil.

#### 3.1.3. Acupoints

In the conducted studies, Baihui (GV20)^[25–27,29–33]^ emerged as the acupoint most frequently utilized, with an incidence rate of 66.7% among the 12 RCTs, followed by Zusanli (ST36)^[24,26,29,31,32]^ with an incidence rate of 41.7%. Furthermore, the inclusion of other acupoints such as Sishencong (EX-HN1),^[26,31,32]^ Yintang (GV24^+^),^[26,27,29]^ Fengchi (GB20),^[26,27,29]^ Shenshu (BL23),^[26,31,32]^ and Neiguan (PC6)^[24,26,33]^ were also observed.

#### 3.1.4. Outcomes

A total of 8 studies^[25–27,29,31–34]^ evaluated the clinical efficacy rate as main outcomes. Six studies^[25,27–30,34]^ assessed the ADAS-cog results. Eight studies^[24,27–29,32–35]^ evaluated the MMSE results. Four studies^[28,32,33,35]^ assessed the ADL results. Two studies^[25,33]^ assessed the BEHAVE-AD results, and 1 study^[30]^ evaluated the MoCA results.

### 3.2. Assessment of risk of bias in included studies

**Figure 2.**
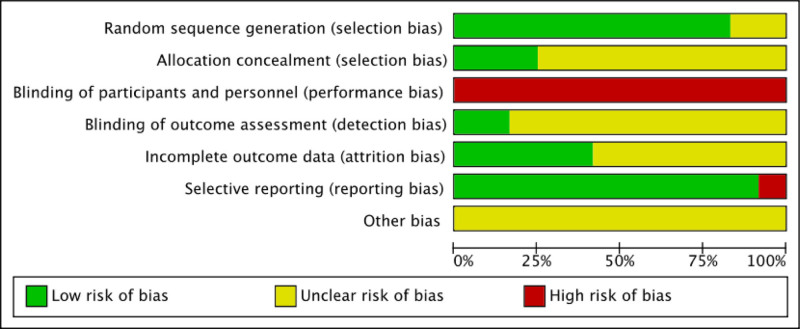
Bar chart of risk of bias.

**Figure 3.**
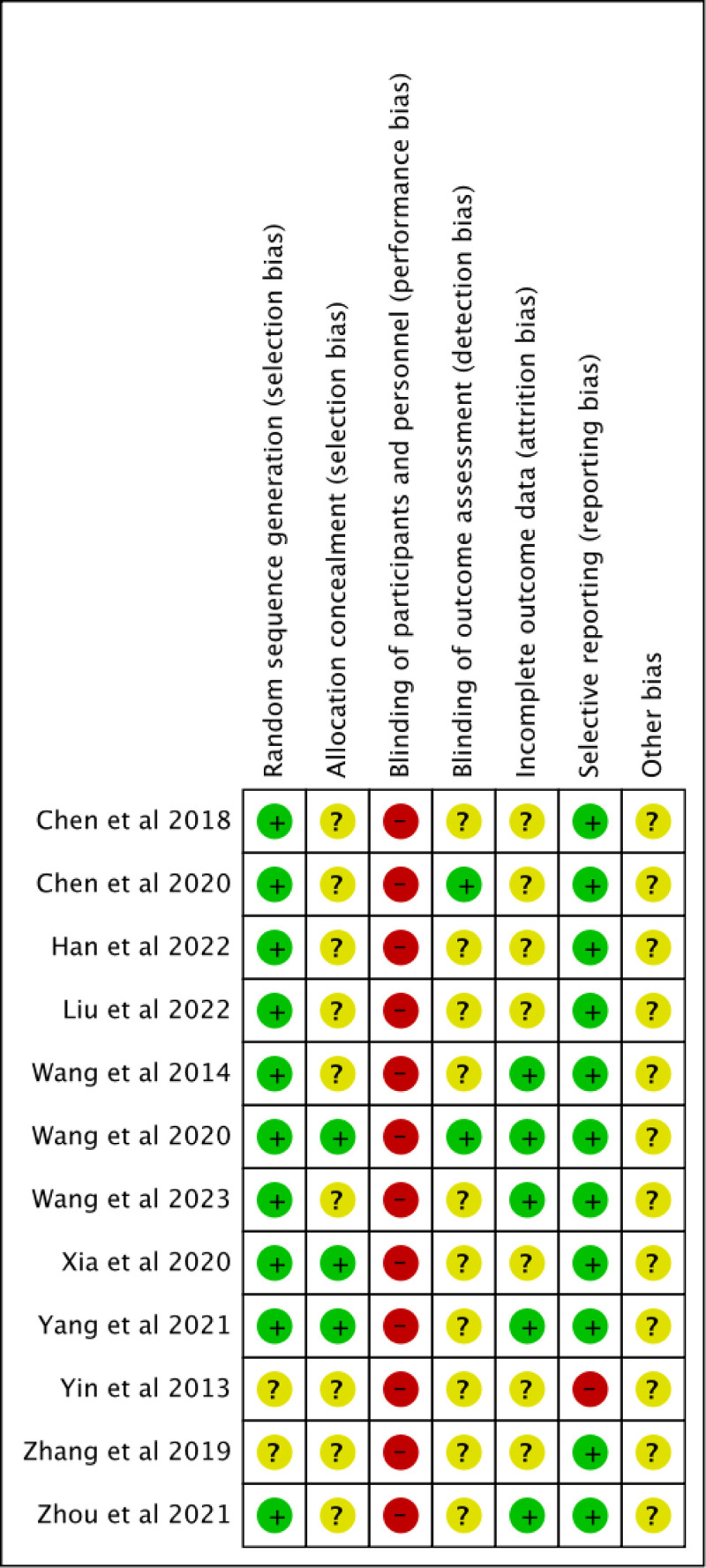
Summary chart of risk of bias.

Figures 2 and 3 show the overall assessment of risk of bias in included studies. All the studies reported randomization. Six studies^[25–27,31,33,34]^ used a random number table while 4 studies^[24,28–30]^ used computer-generated random sequences, and only 3^[28–30]^ of them mentioned allocation concealment. Two studies^[32,35]^ reported the utilization of randomization but did not provide explicit details regarding the specific implementation procedures. None of the included studies blinded the personnel or participants because of the nature of acupuncture. Only 2 studies^[29,31]^ implemented blinding for outcome assessments, while the remaining studies lacked clarity on whether their outcome assessments were truly blinded. It remained ambiguous whether the majority of studies experienced participant attrition, and merely 5 studies^[24,27–29,34]^ reported the inclusion of patients with shedding, thereby potentially introducing attrition bias. The outcomes of most of studies were fully reported, only 1 study^[35]^ conducted safety evaluation, including neurological examination, vital sign examination, laboratory examination and so on, but did not report the results. It was not clear whether there were other biases in all studies.

### 3.3. Outcomes of interventions

#### 3.3.1. Clinical efficacy rate

**Figure 4.**
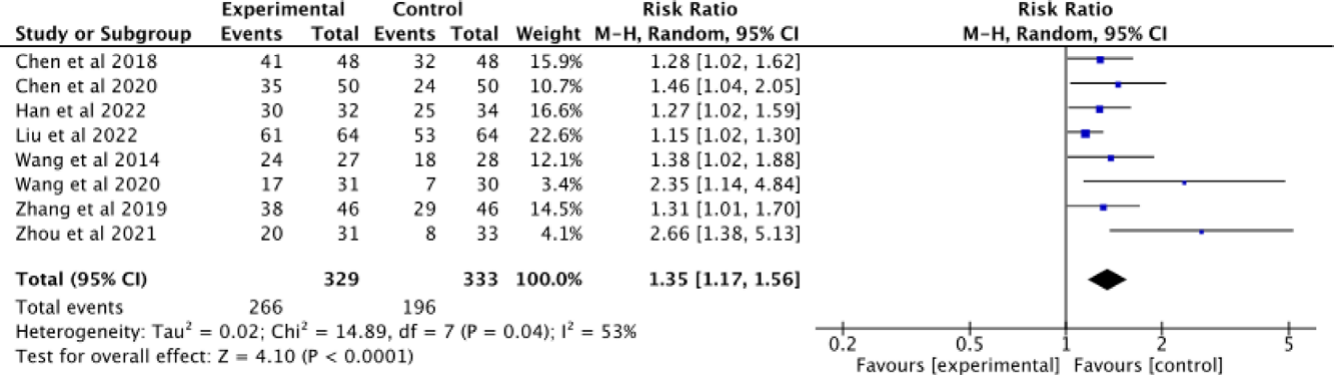
Forest plot of clinical efficacy rate of acupuncture combined with donepezil versus donepezil in the treatment of AD. AD = Alzheimer's disease.

Eight studies^[25–27,29,31–34]^ were included to evaluate the clinical efficacy rate. Figure 4 depicts that these studies exhibited moderate heterogeneity (*P* = .04, I^2^ = 53%), thus a random-effects model was employed for meta-analysis. There was a statistically significant difference observed between the experimental and control groups (RR = 1.35; 95% CI: 1.17–1.56; Z = 4.10; *P* < .0001), suggesting that AD patients who received acupuncture therapy in combination with donepezil exhibited superior clinical efficacy compared to those who solely received donepezil.

Sensitivity analysis was performed by excluding each study 1 at a time to assess the impact of their removal on the results due to statistical heterogeneity observed in the 8 studies. The exclusion of Zhou 2021 study^[27]^ resulted in a significant decrease in heterogeneity (*P* = .26, I^2^ = 23%), which may be attributed to differences in sample size, frequency of acupuncture intervention and follow-up time compared to other studies analyzed. There remained a statistically significant difference between experimental and control groups across all other studies (RR = 1.28; 95% CI: 1.15–1.42; Z = 4.55; *P* < .00001). The marked change in effect size and decreased heterogeneity upon exclusion of this study indicates its influence on result stability.

#### 3.3.2. ADAS-cog

**Figure 5.**
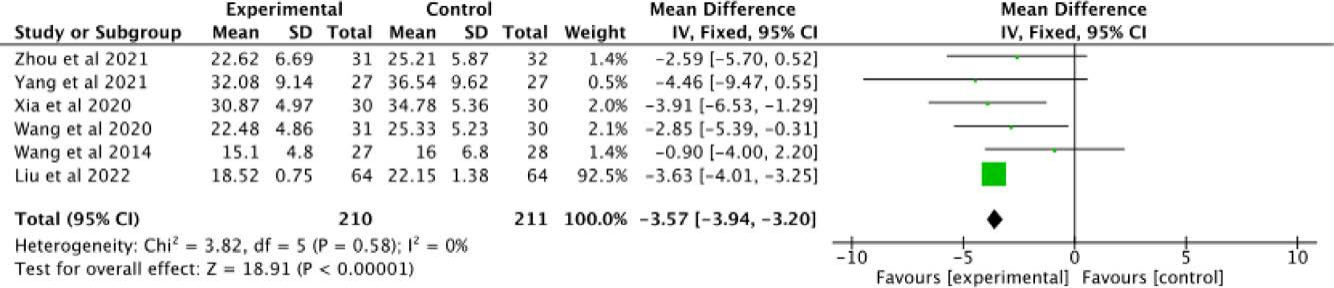
Forest plot of ADAS-cog of acupuncture combined with donepezil versus donepezil in the treatment of AD. AD = Alzheimer's disease, ADAS-cog = Alzheimer's Disease Assessment Scale for Cognitive Capacity.

Six studies^[25,27–30,34]^ were included to assess the results of ADAS-cog. Figure 5 shows that a low level of heterogeneity (*P* = .58, I^2^ = 0%) existed between these studies so a fixed-effects model was used for meta-analysis. The findings revealed a statistically significant difference between the experimental and control groups (MD = -3.57; 95% CI: -3.94 to -3.20; Z = 18.91; *P* < .00001). AD patients who adopted acupuncture combined with donepezil had a superior cognitive performance compared to those who used donepezil alone according to ADAS-cog scores.

#### 3.3.3. MMSE

**Figure 6.**
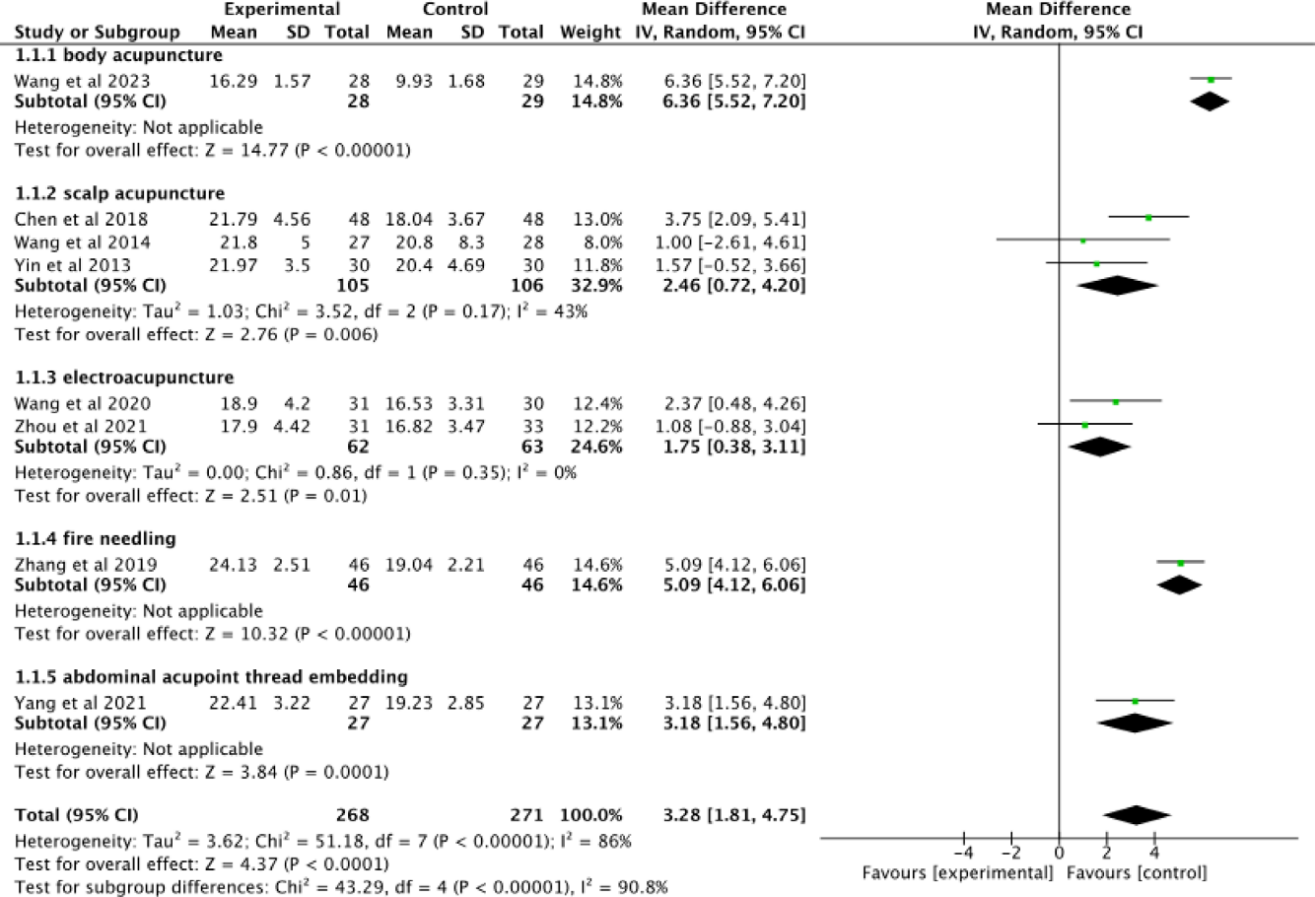
Forest plot of MMSE of acupuncture combined with donepezil versus donepezil in the treatment of AD. AD = Alzheimer's disease, MMSE = Mini-Mental State Examination.

Eight studies^[24,27–29,32–35]^ were included to assess the results of MMSE. Figure 6 shows that a high level of heterogeneity (*P* < .00001, I^2^ = 86%) was observed among these studies, leading us to employ a random-effects model for meta-analysis. There was a statistically significant difference between the experimental and control groups (MD = 3.28; 95% CI: 1.81–4.75; Z = 4.37; *P* < .0001).

Subgroup analysis was conducted based on different acupuncture interventions employed in the experimental groups to enhance comparability and explore potential sources of heterogeneity further. Group 1 employed body acupuncture as the intervention. A statistically significant difference was observed between the experimental and control groups (MD = 6.36; 95% CI: 5.52–7.20; Z = 14.77; *P* < .00001). Group 2 utilized scalp acupuncture as the intervention. These studies exhibited moderate heterogeneity (*P* = .17, I^2^ = 43%) and demonstrated a statistically significant difference between the experimental and control groups (MD = 2.46; 95% CI: 0.72–4.20; Z = 2.76; *P* = .006). Group 3 implemented electroacupuncture as the intervention with less heterogeneity observed among these studies (*P* = .35, I^2^ = 0%). There was a statistically significant difference found between the experimental and control groups (MD = 1.75; 95% CI: 0.38–3.11; Z = 2.51; *P* = .01). Group 4 adopted fire needling. The experimental groups exhibited a statistically significant difference compared to the control groups (MD = 5.09; 95% CI: 4.12–6.06; Z = 10.32; *P* < .00001). Group 5 utilized abdominal acupoint thread embedding. There was a statistically significant difference between the experimental and control groups (MD = 3.18; 95% CI: 1.56–4.80; Z = 3.84; *P* = .0001). Subgroup analysis revealed that different interventions had certain effect on the heterogeneity between the studies. Overall, acupuncture combined with donepezil has a better performance in improving MMSE scores.

#### 3.3.4. ADL

**Figure 7.**
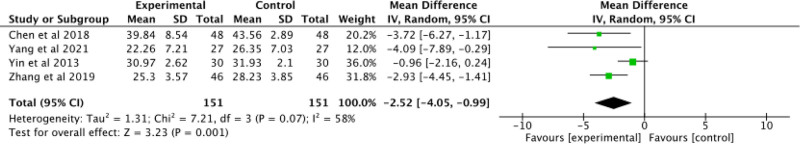
Forest plot of ADL of acupuncture combined with donepezil versus donepezil in the treatment of AD. AD = Alzheimer's disease, ADL = Skill Level on Activities of Daily Living.

Four studies^[28,32,33,35]^ evaluated the results of the ADL. Figure 7 depicts a moderate level of heterogeneity (*P* = .07, I^2^ = 58%) was observed among these studies necessitating the utilization of a random-effects model for meta-analysis. The experimental groups exhibited a statistically significant difference compared to the control groups (MD = -2.52; 95% CI: -4.05 to -0.99; Z = 3.23; *P* = .001). Acupuncture combined with donepezil appeared to be more effective than using donepezil alone in reducing ADL scores.

Due to the statistical heterogeneity observed in the 4 studies, sensitivity analysis was conducted by excluding each study 1 at a time and assessing whether their removal significantly impacted the results. Heterogeneity decreased significantly (*P* = .78, I^2^ = 0%) upon exclusion of Yin 2013 study,^[35]^ which may be attributed to unclear patients’ age and course of disease in that particular study. Notably, there remained a statistically significant difference between experimental and control groups across all other studies analyzed (MD = -3.24; 95% CI: -4.47 to -2.00; Z = 5.14; *P* < .00001). The exclusion of this study resulted in a marked change in effect size and decreased heterogeneity, indicating its influence on result stability.

#### 3.3.5. MoCA

**Figure 8.**
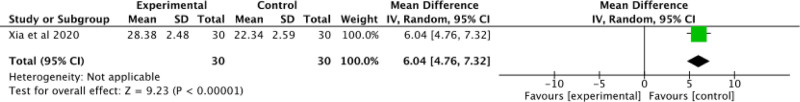
Forest plot of MoCA of acupuncture combined with donepezil versus donepezil in the treatment of AD. AD = Alzheimer's disease, MoCA = Montreal Cognitive Assessment.

One study^[30]^ reported MoCA scores and suggested that the combination of acupuncture with donepezil may lead to a more pronounced improvement in MoCA scores compared to drug therapy alone (MD = 6.04; 95% CI: 4.76–7.32; Z = 9.23; *P* = .00001) (Fig. 8).

#### 3.3.6. BEHAVE-AD

**Figure 9.**
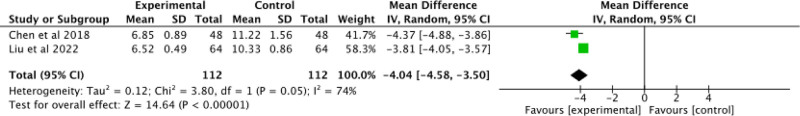
Forest plot of BEHAVE-AD of acupuncture combined with donepezil versus donepezil in the treatment of AD. AD = Alzheimer's disease, BEHAVE-AD = Behavioral Pathology in Alzheimer's Disease Rating Scale.

Two studies^[25,33]^ reported BEHAVE-AD scores and significant heterogeneity (*P* = .05, I^2^ = 74%) was identified among these studies, therefore a random-effects model was utilized. The meta-analysis results indicated that the combined intervention of acupuncture with donepezil could result in a more substantial reduction in BEHAVE-AD scores when compared to drug therapy alone (MD = -4.04; 95% CI: -4.58 to -3.50; Z = 14.64; *P < *.00001) (Fig. 9).

### 3.4. Safety

Six studies^[24,27,28,30,32,35]^ investigated the safety aspects of the trial. One study^[32]^ reported that there were 2 instances of nausea or vomiting in the experimental group with an incidence rate of adverse events at 4.35%. In contrast, the control group experienced 8 cases of adverse events including 2 cases of diarrhea, 5 cases of nausea or vomiting, and 1 case of insomnia. The incidence rate of adverse events in the control group was found to be significantly higher at 17.39%. A significant difference existed between the 2 groups regarding their incidence rates of adverse events (*P* < .05). Another study^[27]^ documented 3 mild adverse reactions in the experimental group and 2 mild adverse reactions in the control group; however, none required any treatment intervention. Three studies^[24,28,30]^ reported no occurrence of any adverse events during the trial period. One study^[35]^ conducted a comprehensive safety evaluation encompassing neurological examination as well as vital sign and laboratory assessments but did not provide final results for these evaluations.

## 4. Discussion

### 4.1. Principal findings

A total of 893 subjects were included of 12 RCTs in this review. All trials included administered donepezil monotherapy in the control group, while the experimental group received acupuncture combined with donepezil. The results of meta-analysis indicated that combining acupuncture with donepezil for AD offered several advantages over donepezil monotherapy in terms of enhancing overall clinical efficacy, improving MMSE and MoCA scores and reducing ADAS-cog, ADL, and BEHAVE-AD scores. Acupuncture combined with donepezil may prove to be a more effective approach for enhancing memory, cognition, and daily living abilities in AD patients.

### 4.2. Mechanism of acupuncture

Acupuncture has demonstrated significant efficacy in enhancing cognitive function which can be seem in changing cognition scale score such as ADAS-cog, MoCA, while maintaining a high level of tolerability and safety among patients with AD.^[36,37]^ These therapeutic effects were also evident in acupuncture combined with Donepezil therapy.^[30]^ Some clinical trials had confirmed that acupuncture combined with donepezil can improve the cognition of AD patients by reducing the levels of amyloid precursor protein and β-amyloid (Aβ) _1–42_,^[28,30]^ reducing serum homocysteine levels^[24,38]^ and modulating the brain activity and functional connectivity.^[16,39]^

Existing studies have revealed that the pathogenesis of AD encompasses Aβ deposition,^[40]^ hyperphosphorylation of Tau protein,^[41]^ central neuroinflammatory response,^[42]^ oxidative stress,^[43]^ and apoptosis^[44]^ among others. Based on previous animal studies, AD model animals could have a better performance in the morris water maze test after receiving acupuncture therapy,^[45]^ the specific intervention mechanisms of acupuncture therapy for AD are as follows: (1) mitigate Aβ production,^[46]^ enhance Aβ clearance,^[47]^ and consequently ameliorate Aβ deposition; (2) suppress the expression of Glycogen synthase kinase-3β^[48]^ and p38 mitogen activated protein kinase^[49]^ proteins to inhibit hyperphosphorylation of Tau protein; (3) modulate microglia^[50,51]^ and astrocyte^[52]^ activation, regulate inflammatory factor release, thereby alleviating central nervous system inflammation; (4) attenuate oxidative stress to mitigate apoptosis of hippocampal nerve cells,^[53]^ thus exerting a neuroprotective effect on hippocampal neurons. In general, acupuncture can exert a favorable influence on AD through various mechanisms.

### 4.3. Limitation of included studies

A certain degree of heterogeneity exists in the studies encompassing MMSE, ADL, MoCA, and BEHAVE-AD. This can be attributed to various factors including diverse intervention methods, disparate treatment durations, distinct acupoint selection criteria, variations in age and disease progression among patients. Furthermore, the quality of each individual study may also exert an influence on the obtained results.

All the 12 RCTs acknowledged the use of randomization, though 2^[32,35]^ did not provide details on the specific method employed. Only 3 studies^[28–30]^ implemented a process of allocation concealment, which could lead to potential selection bias. Due to the nature of acupuncture, none of the included studies blinded the personnel and the participants. All the studies included in this analysis were deemed to have a high risk of performance bias. Only 2 studies^[29,31]^ included in the analysis provided information regarding blinding of outcome assessment, which may lead to potential detection bias. It was unclear whether most studies had lost participants, and only 5 studies^[24,27–29,34]^ reported the presence of shedding patients, which would cause attrition bias. The outcome reports of most studies were complete. One trial^[35]^ mentioned safety evaluation but did not report in the end, which may cause reporting bias. All the RCTs were conducted exclusively in China, with the majority of them being published in Chinese. This could potentially introduce publication bias into our findings. Due to the variety of symptoms and limited documentation on adverse events, conducting a meta-analysis on such events proves challenging, therefore, we have provided a comprehensive description of these occurrences. In general, acupuncture combined with donepezil has fewer adverse reactions and demonstrates higher levels of safety.

Overall, the methodological and reporting quality of the included RCTs were unsatisfactory. There are problems with the description of random sequence generation, allocation concealment, blinding of participants, personnels, and outcome assessors in some studies. The small sample size of most studies and the insufficient number of high-quality RCTs had led to the inability to provide strong evidences. Moreover, the follow-up was inadequate and the long-term data was insufficient. Given the intricate nature of AD, a comprehensive assessment of treatment efficacy necessitates thorough evaluation of long-term outcomes.

### 4.4. Implication for research

The process of randomization and allocation of concealment should be rigorously documented in the future, while more comprehensive descriptions of the randomization methods employed are also provided. It is crucial to ensure data integrity, clarify the ways to deal missing data, and strictly adhere to blinding. In order to enhance the efficacy of acupuncture, it is necessary to consider prolonging treatment sessions and appropriately extending the duration of acupuncture retention. The uniformity of acupuncture points taken in the same experiment should also be ensured. Moreover, conducting a trial comparing acupuncture with sham acupuncture would provide stronger evidence for the effectiveness of acupuncture. More clinical trials with large samples, high quality and in line with international norms are needed to further verify the clinical value and safety of acupuncture combined with donepezil for AD patients, so as to make the conclusions more convincing and provide reliable evidence-based evidences for clinical practices.

## 5. Conclusion

In this study, a systematic review and meta-analysis of included RCTs was conducted to investigate the effectiveness of acupuncture combined with donepezil compared to donepezil monotherapy in the treatment of AD. The findings revealed that acupuncture combined with donepezil demonstrated superior outcomes in improving MMSE and MoCA scores and reducing ADAS-cog, ADL and BEHAVE-AD scores among AD patients when compared to donepezil monotherapy. Moreover, the combination therapy exhibited higher clinical treatment efficiency, suggesting its potential for enhancing cognitive ability and quality of life in AD patients. This study serves as a foundation for integrating traditional Chinese medicine with western medicine for treating AD while enriching the clinical intervention strategies involving drug–non-drug combinations. However, it is imperative to conduct more large-scale and high-quality RCTs in order to establish more definitive conclusions regarding this therapeutic approach.

## Author contributions

**Conceptualization:** Yue Wu, Jian Pei.

**Data curation:** Yue Wu, Yijun Zhan, Jian Pei.

**Formal analysis:** Yue Wu, Wenyan Zhu.

**Investigation:** Yue Wu, Wenyan Zhu.

**Methodology:** Yue Wu, Yijun Zhan, Jian Pei.

**Project administration:** Jian Pei.

**Supervision:** Jian Pei.

**Software:** Yue Wu, Yijun Zhan.

**Validation:** Jian Pei.

**Visualization:** Jian Pei.

**Writing – original draft:** Yue Wu.

**Writing – review & editing:** Yue Wu.
